# Discerning Pig Screams in Production Environments

**DOI:** 10.1371/journal.pone.0123111

**Published:** 2015-04-29

**Authors:** J. Vandermeulen, C. Bahr, E. Tullo, I. Fontana, S. Ott, M. Kashiha, M. Guarino, C. P. H. Moons, F. A. M. Tuyttens, T. A. Niewold, D. Berckmans

**Affiliations:** 1 M3-BIORES—Measure, Model & Manage Bioresponses, KU Leuven, Leuven, Belgium; 2 Department of Health, Animal Science and Food Safety, Università degli Studi di Milano, Milan, Italy; 3 Livestock-Nutrition-Quality, KU Leuven, Leuven, Belgium; 4 Departement of Animal Nutrition, Genetics and Ethology, Laboratory for Ethology, Ghent university, Merelbeke, Belgium; 5 Institute for Agricultural and Fisheries Research (ILVO), Animal Sciences Unit, Melle, Belgium; University of Salamanca- Institute for Neuroscience of Castille and Leon and Medical School, SPAIN

## Abstract

Pig vocalisations convey information about their current state of health and welfare. Continuously monitoring these vocalisations can provide useful information for the farmer. For instance, pig screams can indicate stressful situations. When monitoring screams, other sounds can interfere with scream detection. Therefore, identifying screams from other sounds is essential. The objective of this study was to understand which sound features define a scream. Therefore, a method to detect screams based on sound features with physical meaning and explicit rules was developed. To achieve this, 7 hours of labelled data from 24 pigs was used. The developed detection method attained 72% sensitivity, 91% specificity and 83% precision. As a result, the detection method showed that screams contain the following features discerning them from other sounds: a formant structure, adequate power, high frequency content, sufficient variability and duration.

## Introduction

Animal vocalisations can contain information such as signalling threats [1], choosing mates [2] or alerting infants for suckling [3]. In case of livestock animals, information contained in vocalisations or other animals sounds could serve as valuable information for the farmer. A very good example is the rich vocal repertoire of pigs [4–6]. For instance, high frequency calls of pigs have already been linked to stressful situations [7]. Moreover, animal sounds such as coughs could be linked to respiratory diseases and thus to their welfare [8,9]. Therefore, vocalisation could be useful for assessing the animal’s condition. Furthermore, the use of technology to monitor these vocalisations opens new possibilities as they can be monitored automatically and continuously. In the past numerous research studies on pig vocalisations in stressful situations have focussed on analysing high frequency calls.

In these studies on high frequency vocalisations during different situations were analysed such as diverse castration practices [10–12], cold [13] or warm [14] temperatures. Other examples were the simulated crushing of piglets [15] or an electric shock or anticipation to the electric shock [16]. For the remainder of the paper, these high frequency calls are called screams. They are defined as vocalisations containing considerable high frequency content and having a larger amplitude than other vocalisations [17]. For the difference between screams and squeals the reader is referred to literature [18].

These previous studies had one limitation, they focused on analysing screams while ignoring other sounds present in a pig barn. Two exceptions conducted analysis on three sound types: screaming, squealing and grunting sounds [18,19]. However, these are not the only sounds present in a pig barn. For instance other vocalisations such as barks, coughs or environmental sounds such as the automated feeder, drinking nipple and the farmer are present. For some of these sounds such as coughs [20] and barks [21], separate studies have been performed in analysing them. In general, a new approach is needed that identifies the features that discern screams from all other sounds in a pig barn. These other sounds do not have to be identified in this new approach.

When discerning screams in this new approach, the initial condition requires features with physical meaning [22]. Features such as loudness, duration, fundamental frequency and formant structure [23] are defined as sound parameters which are simply interpreted and physically related to vocalisation. However, speech processing terms such as autoregressive or cepstral coefficients [24], are generally much harder to interpret.

The need for rigourous classification with explicit rules is the next condition for identifying screams. Such rules comprise a set of readily interpretable requirements. For instance, a decision tree with conditions has such rules. An Artificial Neural Network (ANN), however, gives little explicit information about the decision making [25]. For example, ANN can be used for automated stress vocalisation detection of pigs which is called STREMODO [26,27]. However, by using an ANN and autoregressive coefficients this method was unable to interpret sound features.

Using features with physical meaning and explicit rules as explained in previous two paragraphs offers the possibility to develop an automated scream detection method. The advantages of this approach over STREMODO [26] are that the results can be interpreted and that the approach can be adapted online to changing situations. (1) Our new approach offers the possibility to interpret different classes of screams. Moreover, (2) the rigourous classification can be adapted to each specific situation. For instance, during feeding time, more screams are expected due to competition between animals and this does not necessarily indicate serious stressful situation. While screams detected during night time would indicate serious stressful situation. So during feeding time, only screams indicating serious stressful situation should be detected. Such screams could have more high frequency content [11] or have a longer duration [6]. While during night time every scream should be detected.

The purpose of this study is to investigate what sound features define a pig scream as a pig scream and how they differ from other sounds in a pig barn. To achieve this goal an automated scream detection method was developed based on sound recordings made in a real scale experimental pig barn. This method is supposed to discern screams from other sounds present. These other sounds are not named but represent all sounds originally detected by this method. Moreover, this method should discern them continuously which means once every second. To identify the relevant sound features defining a pig scream a detection method which followed two conditions was constructed. First the calculated features should have a physical meaning and secondly the classification should be made with explicit rules, in order to interpret why a sound is considered a scream. Moreover, special attention during classification was given to features which described the formant structure of screams. Formants are the different spectral peaks in the frequency spectrum of the human voice and are also present in pig screams.

## Materials and Methods

### Animal and Housing

**Fig 1:**
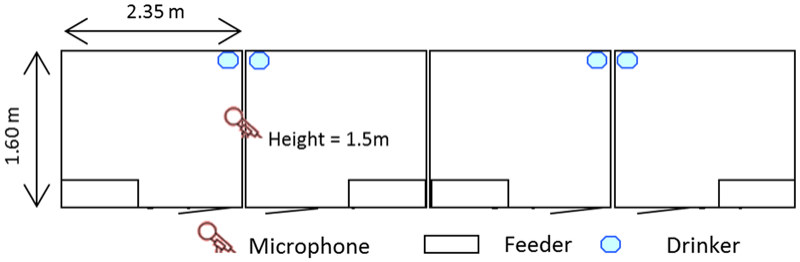
Ground plan of the pig compartment. Each pen had six animals, one feeder and one drinker. One microphone recorded the sound.

Two trials were conducted and 24 grower pigs were used in each trial. The animals Rattlerow Seghers x Piétrain Plus, were housed at Agrivet research farm, Merelbeke, Belgium. After the battery period, they were divided into four groups of six animals (three gilts and three barrows) and each group was assigned to a pen (Fig 1). Each pen (1.60m x 2.35m) had a fully slatted concrete floor with one feeder space and one nipple drinker. The pens were located in the same compartment and were separated from each other with 1m high solid walls. So physical contact between pigs of adjacent pens was made impossible but they could still hear each other. There was ad libitum access to feed (commercial grower diet) and water during the experiment. Pigs had a timer-controlled 12-hour light period from 07:00 h to 19:00 h. The average weight of the pigs was 20.9kg (SD = 2.1) at start and 32.2kg (SD = 3.8) at end of the first trial and 31.5kg (SD = 3.4) and 43.0kg (SD = 5.5) respectively, in the second trial. The average temperature during the trials was 24.0°C (SD = 1.2). The experiment was approved by the Ethical Committee of the Faculty of Veterinary Medicine at Ghent University (EC2012/125).

### Experiment and Data Collection

Each trial lasted 15 days in which two treatments, experienced as stressful by pigs were applied. Prior to each trial pigs had 7 days of adaptation to their new environment. During the trial, on day six, the animals from two randomly chosen pens (P1 and P2) were mixed between 7:00h and 8:00h. For this purpose, three animals of P1 were exchanged with three animals of P2. On day eleven, P1 and P2 were subjected to feed deprivation which started at 12:00h and ended 24 hours later.

The sound data were recorded with a microphone (C-4 Small Diaphragm Condenser Mic, Behringer, Germany) at a height of 1.5 m and a sound card (Delta 1010LT, M-audio, Cumberland, United States) with a precision of 16 bit and a sampling frequency of 22050 Hz. The microphone was positioned as seen in Fig 1, so it recorded sounds of all four pens. In total 720 hours of sound data were collected.

One remarkable situation occurred during the recordings. For all hours our compartment was acoustically separated from the neighbouring compartment. Except one hour when most pigs in this neighbouring compartment were screaming at the same time. The sound power was loud enough to be heard through the separating wall. This occurrence was considered when developing the algorithm.

### Labelling of Sound Data

In order to develop a classifier, i.e., a system to classify pig vocalisations as screams, a reference data set is needed. Because the collected sound possessed screams but it carried no information when screams occurred. The reference was built via labelling by a human observer, who indicated the beginning and end of each scream, using the computer program Adobe Audition (Adobe Systems, San José, US) [28,29]. This human observer, experienced in labelling pig vocalisation, labelled 7 hours of sound data. These hours were chosen randomly except one. This hour contained the vocalisations in which pigs regained access to the feeder after the second stressful treatment. In this paper a distinction is made between the first 6 hours and the last hour. The former consist of 312 screams and the latter consists of 38 screams. Moreover, to assess the labelling performance, the human observer labelled the same 10 minutes on two different occasions. However, this person was unaware of this. The correlation between these labelled files was calculated to assess if this person labelled consistently. This calculation was based on literature for pig stress vocalisations [26]. A correlation of 0.83 (P<0.001) was achieved which was deemed sufficient.

### Classifier Overview

**Fig 2:**
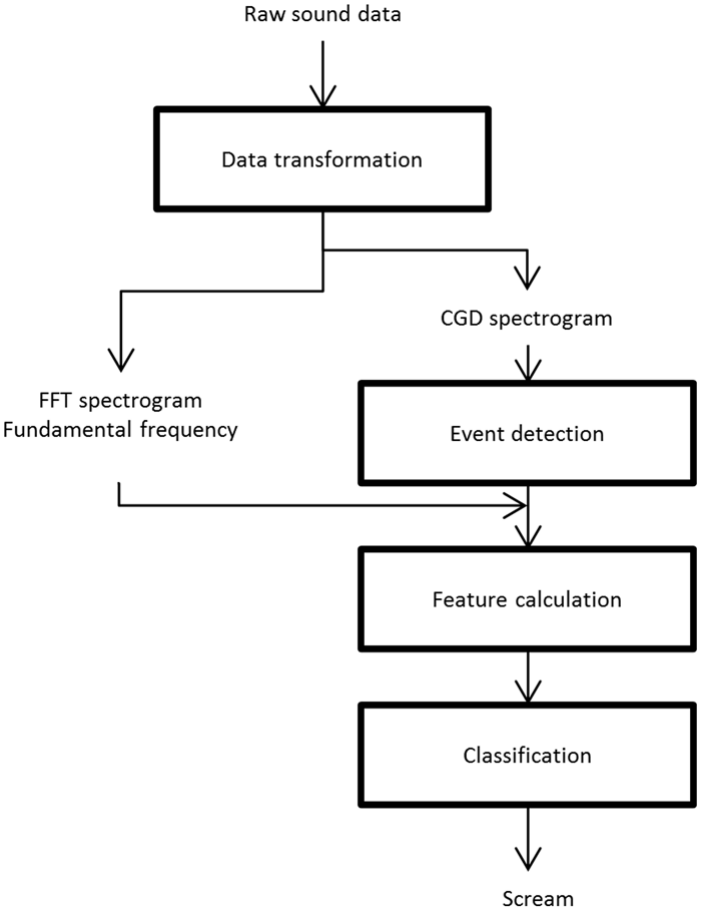
Overview of the classifier elements. The rectangles represent the four parts. The raw data is transformed into an output that indicates if a scream is present. (CGD = Chirp Group Delay, FFT = Fast Fourier Transform)

The classifier consisted of four parts: (1) the data transformation, (2) the event detection, (3) the feature calculation and (4) the classification. Fig 2 shows the scheme of the classifier. In the following paragraphs different elements of the classifier will be explained and their combination will be discussed.

#### Data transformation

To obtain different frequency information, the sound data were transformed using three techniques: (1) the Fast Fourier Transform (FFT), (2) the Chirp Group Delay (CGD) [30] and (3) the fundamental frequency calculation. The FFT analysed high frequency content and CGD analysed formant structure. The fundamental frequency provided the longest periodic pattern in the sound, which in human vocalisation is produced by the glottis. The implementation originally used for calculating fundamental frequency of pig coughs was adopted [31].

**Fig 3:**
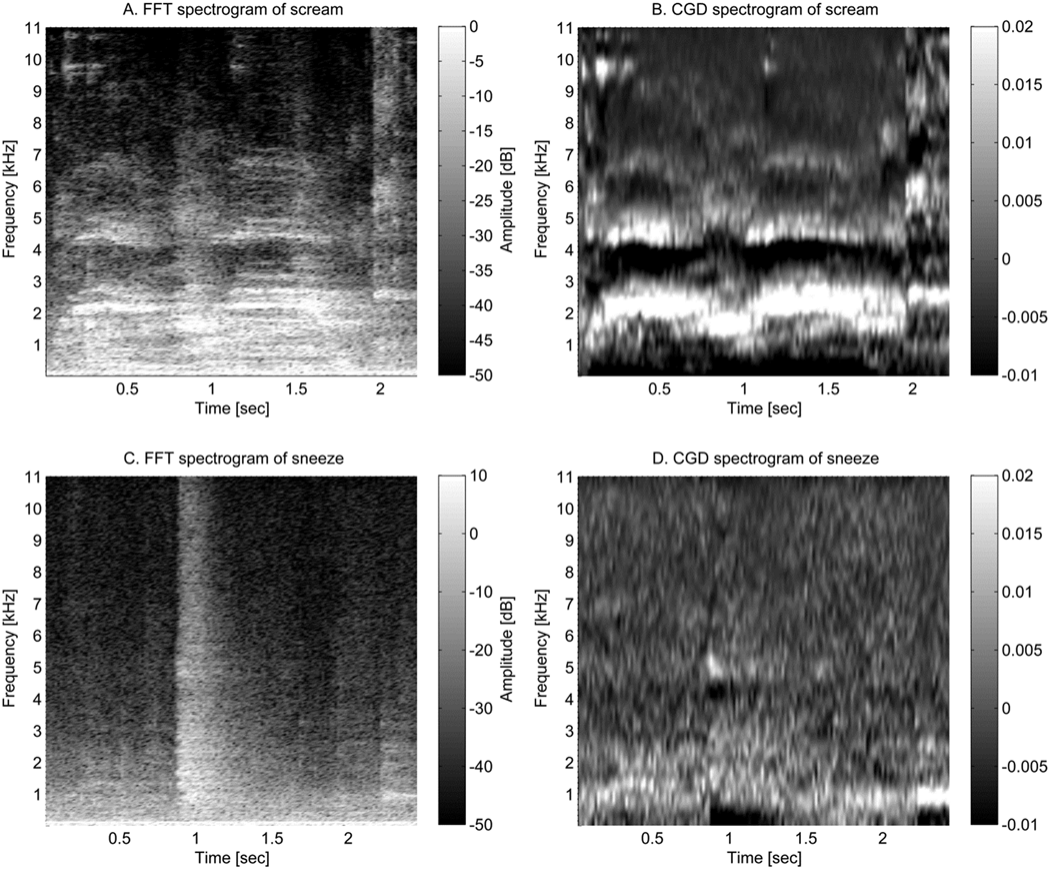
The FFT spectrograms and CGD spectrograms. The upper two figures show the same screams, the lower figures show the same sneeze. The left figures depict the spectrogram made from FFT while the figures on the right side depict the spectrogram made from CGD. The formant structure of a scream is visible (B). These formants are the whiter values in the CGD spectrogram. It is not straightforward to find the same structure in the FFT spectrogram. Because the difference between the formant value and the surrounding values is much bigger relative to the maximum and minimum values of the CGD compared to the FFT

In order to calculate these transformations, sound data was divided into 30ms hamming windows [32] with a 15ms overlap. This duration was chosen similar to speech analysis in which 20-40ms windows are used.[33]. Calculating the transformations on each window provided time frequency information. An example is presented for both FFT and CGD in Fig 3. To further reduce FFT and CGD data, the frequency resolution was lowered into 24 Mel-spaced frequency bands. This experimental scale is used to resemble human perception of sound frequency, particularly fundamental frequency [34].

#### Event detection

The event detection is based on a method for detecting sound events needed for human cough detection [35]. This method adopts two thresholds. One threshold detects peaks in the sound data while the second threshold detects the starting and ending time of the peaks. However, the peaks were found in the standard deviation of the sound data while in the current study, the peaks were found in feature 6 from Table 1. This feature 6 will be described in following paragraph. This feature was chosen because it detected 84% of the labelled screams resulting in 261 screams and only 4552 other sounds. These 261 screams were found in 231 sound events. This means that several sound events consisted of multiple labelled screams.

**Table 1:** The features used by the scream detection algorithm.

Feature category	Feature nr	Feature description
**Event power**	1	Mean of the spectrogram
**High frequency content**	2	Mean of the 12 higher frequencies in the FFT spectrogram
	3	Fundamental frequency
**Formant structure**	4	Maximum value of CGD spectrogram
	5	Number of values in CGD spectrogram above a threshold
	6	Squared error on line fit through mean vales of CGD spectrogram
	7	Third FFT value from mean values CGD spectrogram
	8	Third FFT value normalised by DC value from mean values CGD spectrogram
**Event variability**	9	Standard deviation of CGD spectrum
**Event duration**	10	The duration of the sound event [seconds]

#### Feature calculation

A total of 10 features were calculated from the data transformations for each sound event. These can be ordered into several categories as shown in Table 1. These categories assessed the sound power, the high frequency content, the formant structure, the variability and the duration of the sound events. The aim was to give these categories a physical meaning that can be interpreted easily.

The first feature category is the power and has only one feature. This feature is calculated from the mean value of the FFT spectrogram. Screams are one of the louder sounds in a pig barn and this feature considered this. This feature is less stable because the distance between the animal and the microphone is variable, meaning the value changes. A solution would be to determine the ratio of the sound power to the medial level of all spectra [19].

The second category calculated two features that examined the higher frequency content. The power of the higher frequencies was calculated by taking the mean value of the twelve highest frequencies from the FFT spectrogram and consequently the mean over all windows belonging to the scream. The second feature was the fundamental frequency of pig screams which was already calculated in the data transformation.

**Fig 4:**
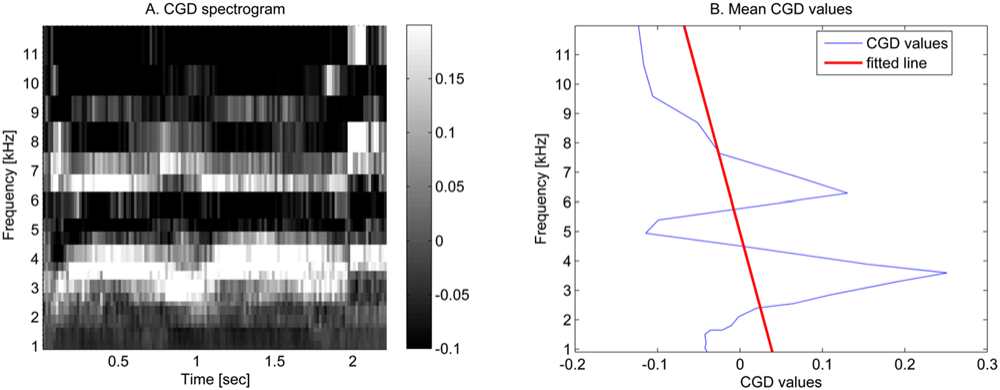
The Mel CGD spectogram. The left figure (A) shows the CGD spectrogram with Mel frequency resolution. The right figure (B) shows the corresponding mean values of each frequency value from the Mel CGD spectrogram. This figure further shows the straight line fitted trough these mean values.

The formant structure was the third feature category and contained the most features. As seen in Fig 3, screams exhibit a formant structure that is visible in the CGD spectrogram and this feature will therefore be used in the third category. The first two features were directly calculated from the CGD spectrogram. With these features the maximum value and the amount of values higher than a threshold was assessed. This threshold was calculated with the technique described in the next section ‘Classification’. The three other formant structure features required the mean values of each frequency value over all time windows of the event. These are shown by the CGD values in Fig 4b. For the third feature a straight line was fitted through these values. The squared error between this line and the mean values was minimised. This resulting squared error was defined as the third feature because a line cannot resemble a formant structure and thus the squared error value of a scream will be higher. For the fourth and fifth feature the formant structure was interpreted by applying an FFT on these mean values. This is the same technique as used in the data transformation paragraph but now applied on different data. As sounds with a formant structure have more fluctuating values compared to other sounds (for instance in Fig 3) they will have larger values at higher frequencies compared to the zero frequency.

Within the fourth feature category, the event variability in the CGD spectrogram was determined by calculating the standard deviation values. For instance the CGD of screams vary more than other sounds. For comparison the spectrogram of a sneeze is also given in Fig 3. The last feature category is the sound event duration. This was calculated from sounds found in the event detection.

#### Classification

Before the classification a preselection of sound events was made based on the labelled data. An event could be a scream if its duration (feature 10) was longer than 0.4s. This threshold was experimentally defined, based on the labelled data. While looking closer at the data it was discovered that screams belonged to the higher values of each feature. However, it also appeared that not every scream had high values for each feature. For example, some screams had a long duration but a low sound power while others were short but had a high sound power.

To cope with the demand for a classification with explicit rules and with the two facts discovered in last paragraph, a threshold was determined for each feature. This threshold split the dataset in two per feature and was made in the same way as a classification tree when splitting by using the Gini Diversity index [36,37]. This index measured the purity of two datasets. Purity is a measure which indicates the homogeneity of a dataset. Several thresholds in ascending order were applied and consequently the threshold that maximised the purity was chosen. Because not every scream had high values for each feature, thresholds were combined into a simple voting system [38]. Each feature had one vote to decide if a sound belonged to a scream or not. These vote were later summed together.

Having a classifier with votes offers the possibility to make the classifier adaptive. As discussed in the introduction, during feeding time, screams related more to serious stressful situation should be detected. This could mean screams with a higher vote or screams for which one threshold was increased such as the duration [6]. While during night time, screams with a lower vote could be detected instead.

#### Construction of the classifier

The four different parts: data transformation, event detection, feature calculation and classification were combined as depicted in Fig 2. The data were first transformed from time series into time-frequency representations in order to calculate the events and feature values. Afterwards the event detection constructed the sound intervals that possibly contained a scream. Subsequently the features were computed for these sound intervals of interest. Finally, the classification decided if a sound event was a scream.

#### Validation of the classifier

The classifier was constructed from six of the seven hours of labelled data. The sound events were extracted from these six hours with the event detection as described in a previous paragraph. This resulted in screams and other sounds. The other sounds comprised of other vocalisations and other environmental sounds. Two third of these sound events were randomly selected as training set for construction of the classifier. The remaining one-third was selected to validate the classifier. The criteria to assess the results were sensitivity, specificity, precision and the Receiver Operating Characteristic (ROC curve) [39].

$$\;sensitivity=\frac{numberoftruepositives}{numberoftruepositives+numberoffalsenegatives}$$ (1)

$$specificity=\frac{numberoftruenegatives}{numberoftruenegatives+numberoffalsepositives}$$ (2)

$$precision=\frac{numberoftruepositives}{numberoftruepositives+numberoffalsepositives}$$ (3)

The ROC curve plots the True Positive Rate (TPR = sensitivity) versus the False Positive Rate (FPR = 1—specificity) for various number of required votes. The ROC curve’s purpose was to determine the number of votes required to classify a sound as a scream. Because sensitivity and specificity could be easily compared in this curve.

A total of 10 minutes of the 7^th^ hour was used for a different validation. This validation calculated the correlation between labelling result and algorithm result. In this way it provided an indication if labelled screams followed the same pattern as found by the algorithm. This correlation was calculated as proposed by Schön et al. [26] for stress vocalisations. The remaining 50 minutes of the last hour were not used as they consisted of only 4 labelled screams as opposed to the 34 labelled screams in the chosen 10 minutes.

#### Assessing the defining features of a scream

The resulting classification structure allowed assessing each feature and the corresponding threshold for their share in the final vote. For instance, the percentage of true positives that satisfied a specific feature threshold was calculated. Or in other words, the percentage of true positives that received a vote from this specific feature threshold. This allowed explaining which feature thresholds contributed more to the scream detection. This analysis was expanded to two other sets: all labelled screams and all other sounds, providing the TPR and FPR per feature threshold. These analyses were applied to the combined training and validation dataset.

## Results

**Fig 5:**
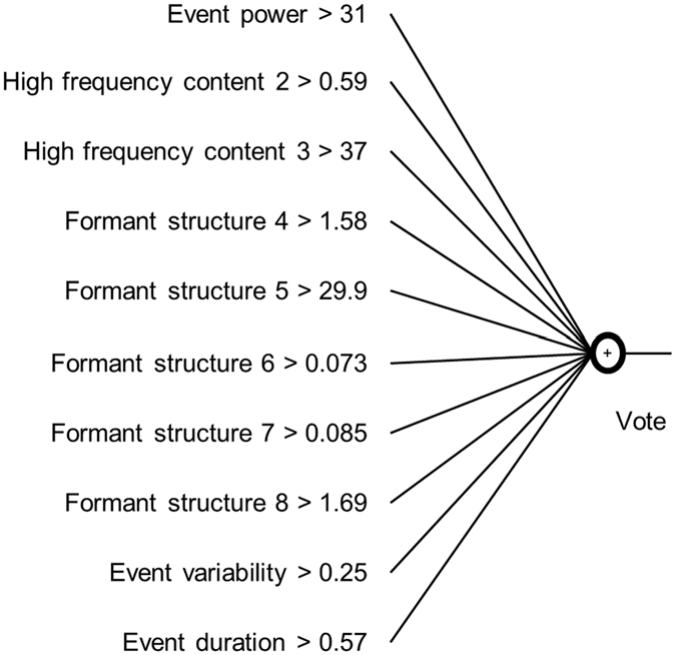
The resulting classification. These were the found thresholds for each feature after applying the gini index to all sound events in the traing dataset. Each line resembles on feature with acompagning threshold. When an event’s feature value was above the threshold value, the sound event received one vote. All ten votes were subsequently summed together for one sound event.

In accordance with the event selection 4783 sound events were found. A total of 231 events agreed with screams found by human labelling. After preselection as described in the section about classification 563 sound events remained. A total 213 events contained labelled screams. These 563 sound events were subsequently subjected to the final classification as shown in Fig 5.

**Fig 6:**
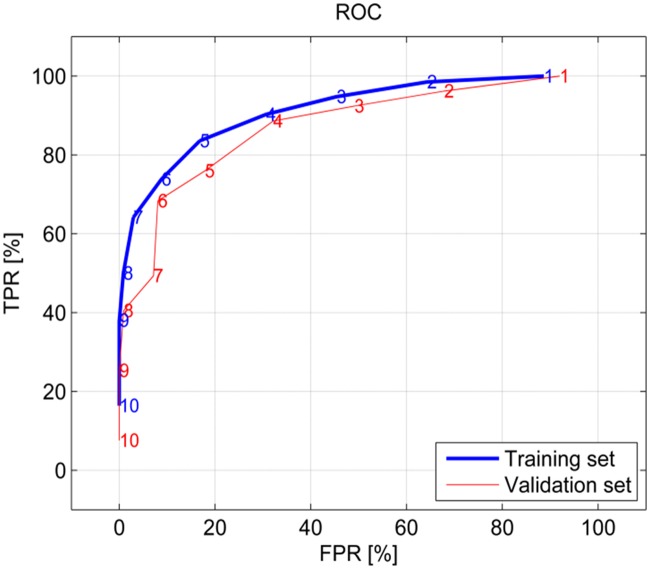
The two ROC curves. They showing the True and False positive Rates (TPR and FPR). The numbers on the plots give the minimal required votes for the training and validation set.

Fig 6 depicts the ROC-curve for the various numbers of votes required for classification as a scream. According to ROC the training set had consistently higher sensitivity (or TPR) values than the validation set. On average it was 0.07 (or 7%) higher. Furthermore, the desired sensitivity and specificity could be chosen based on this curve. The remainder of the results were calculated with six as the minimal number of votes required. The reason for choosing six is explained in the discussion.

As depicted in Table 2 when choosing six as minimal number of votes, the sensitivity of the training set was higher than the validation set but the specificity and precision was lower. Moreover the correlation between the labelled data and the screams found by the algorithm was 79.95% (P<0.001).

**Table 2:** The sensitivity, specificity and precision.

	Training set	Validation set	All sets
**Number screams**	134	79	213
**Number of others**	238	112	350
**Sensitivity**	73.9%	68.5%	71.8%
**Specificity**	91.2%	92.0%	91.4%
**Precision**	82.5%	85.7%	83.6%

The sensitivity, specificity and precision of both sets separately and combined when six votes are required. The number of others refers to sound events that are not screams.

Furthermore the share of each feature and corresponding threshold is indicated in Table 3. This allowed to discuss the importance of each feature in defining a scream. From this table it became clear that the percentage of true positives (TP) for all feature thresholds except for feature ‘Formant structure 4’ had percentages higher than 75%. ‘Formant structure 4’ had 30.1%. The percentages of all scream events performed poorer than the TPs because the True Negatives (TN) were included in the computation. Overall every percentage was higher than 50% except again for feature ‘Formant structure 4’. The third row gives the same analysis for all the other sounds. Every value was lower than 50% and remarkably ‘Formant structure 4’ also scored the lowest value with 3.7%. The different percentages of feature ‘Formant structure 4’ are caused by applying the Ginny Diversity index [36] in the classification. This index maximises the purity and this resulted in 21.6% and 3.7% of screams and other sounds, respectively. It could be that ‘Formant structure 4’ is feature for a specific class of screams and not in general of pig screams.

**Table 3:** Performance of the features.

	TP	Screams (TP+FN)	Others (TN+FP)
**Event Power**	93.50%	85.40%	24.50%
**High frequency content 2**	74.50%	59.60%	13.40%
**High frequency content 3**	85.60%	78.40%	32.30%
**Formant structure 4**	30.10%	21.60%	3.70%
**Formant structure 5**	86.90%	66.20%	10.60%
**Formant structure 6**	93.50%	79.80%	44.30%
**Formant structure 7**	93.50%	82.20%	45.10%
**Formant structure 8**	86.90%	69.50%	25.10%
**Event variability**	75.20%	57.30%	12.60%
**Event duration**	91.50%	85.40%	52.00%

The percentage of True Positives (TP), all screams and other sounds that satisfy each feature’s threshold. Or in other words the percentage of TPs that receive a vote from each threshold.

## Discussion

The purpose of this paper was to investigate what sound features define a pig scream as a pig scream and how they differ from other sounds in a pig barn. To achieve this, a classifier using features with physical meaning was constructed. This ability is one advantage of the method compared with STREMODO [26]. A total of 10 features were developed which belonged to 5 categories: The power, the higher frequency content, the formant structure, the variability and the duration of each sound event. Subsequently a classifier based on explicit rules was developed and was depicted in Fig 5. The performance of the classifier is first examined, the adaptive ability of the classifier is shortly discussed and afterwards the discerning sound features are discussed.

### Performance of the classifier

Performance of the classifier is displayed in Fig 6. Increasing the number of minimal votes required, decreased the TPR and increased the FPR. Because other sound events, such as coughs or sneezes are usually more prevalent in pig barns, a high specificity was desired, whereas sensitivity was of less importance. Six was, therefore, selected as the minimal number of votes as this gave a specificity higher than 90%. Moreover, this gave eventually 92% specificity, 69% sensitivity and 86% precision for the validation set (Table 2).

The scream detection method performance could be compared with a system called STREMODO [26]. Although there are several differences such as the target vocalisation: screams compared to stress vocalisation; a cautious comparison is made. The sensitivity and specificity obtained by STREMODO, 99.3% and 98.6%, respectively were better than our method. Moreover, their results were obtained from sounds recorded in a noise-reduced chamber with less sound reflections [40]. Our results, however, were obtained in a real scale experimental pig barn with additional sounds, such as pigs playing with chains. In reality, there will be other sounds present during screaming sounds. Another reason for our lower sensitivity and specificity is the classifier’s complexity. STREMODO used a complex ANN with 194 perceptron and 4 layers while we used 10 thresholds and a voting system.

Another way to compare STREMODO with our developed algorithm is by calculating the correlation between our algorithm and the labeller for 10 minutes. For STREMODO this feature was calculated in commercial pig barn hence this is comparable to our set-up. Our method achieved a correlation of 0.80 (P<0.001), which was comparable with the correlation obtained by STREMODO (0.84; P<0.001) in which six experts labelled pig screams.

### Adaptive ability classifier

One of the mentioned advantages of this new approach over STREMODO [26] was the adaptive ability of the automated detection method. The developed detection allows for an adaptive threshold both on the number of votes as for each of the ten features. For instance, it is very easy to increase a threshold on the duration feature during feeding time so that calls associated with serious stressful situation are detected [6]. Or to decrease the number of required votes during the night time to certainly detect all screams. This would be possible as the number of other sounds also decreases during the night. In general, the sensitivity, specificity and precision should always be considered when adapting these thresholds. However, this was not developed in this study as more labelled sound files during different situations were necessary than currently available to validate this.

### Features defining a pig scream

The goal of this study was to define what features make a pig scream a pig scream. The percentage of screams satisfying each threshold in the classifier were given (Table 3). Consequently, identifying the defining scream sound features in our experimental pig barn was now possible. Generalisation to other pig barns should be done with caution as for instance, sound from an automatic feeder may be present. In following paragraphs each feature category will be investigated.

(1) A scream should have a certain power (feature 1). This is evident as screams are one of the louder sounds in a pig barn. This corresponded with literature in which the mean relative sound energy of screaming was 15dB higher than grunting and squealing [19]. According to Table 3, the power was one of the most defining sound features of a scream. In total 93% true positives received a vote from this feature. While only 24% of the found other sounds in this dataset conformed to this threshold. Furthermore among all features, the difference in percentage between screams and other sounds was highest for this feature, attaining 61%.

(2) Screams have more higher frequency content than other sounds as seen in Table 1. The two features that described the higher frequency content showed this (Mean of the 12 higher frequencies in the FFT spectrogram and fundamental frequency; Table 1). This corresponds with literature in which the peak and main frequencies of screams were significantly higher than grunts [18]. However, they did not consider every sound in a pig barn while this paper considered all sounds present during the labelled hours for this specific pig barn. According to Table 3 75% and 85% of the true positives received votes from feature 2 and 3, respectively.

(3) A scream should have a formant structure. In this study, there were five features assessing the formant structure. These features did not specify the exact values of the formants as in literature [41] but attempted to give an indication if a formant structure was present. Furthermore, a data representation called Chirp Group Delay (CGD) [30] was applied for the first time on animal vocalisations while previous studies applied LPC coefficients to represent this structure in stress vocalisations [26,41]. Moreover, other sounds present in a pig barn such as barks [21] and coughs [42] were shown to possess certain formant structure but these sounds were not included in this research paper. The performance of the first formant feature (maximum value of CGD spectrogram) was poor at first sight according to Table 3. Only 30% of the TPs received a vote. According to the last row, however, only 4% of the other sounds received votes from this feature. This feature had, therefore, low sensitivity but high specificity. The other four features accounted for at least 87% of the TPs and except for feature 6 and 7 the other sounds received low percentage of votes. In general they performed well on separating screams from other sounds.

(4) Screams should vary considerably (Standard deviation of CGD spectrum; Table 1), meaning that feature 9 should be higher than 0.25 as seen in Fig 5. Because 75% of the TPs and only 13% of the other sound received votes, this indicated a defining sound feature of screams.

(5) Finally, screams should possess a minimal duration. Because before classification, the sound events shorter than 0.4 seconds were omitted. In accordance with this preselection 563 out of 4783 sound events were removed based on duration. Furthermore, after classification 85% of the produced screams had a longer duration than 0.57s compared to only 52% of the other sounds as seen in Table 3. This is in agreement with literature on young pigs in which longer calls were more associated with negative situations [6] and in which scream duration was significantly longer than grunts or squeals [19]. However, in literature on older pigs such as sows, calls which were not screams were found to be longer than 1s [43]. Moreover, in literature screams were found to have a duration between 0.3s and 3s [5] or on average 1s [17] while pig coughs had an average duration of 0.43s or 0.67s for non-infectious and infectious coughs, respectively [44].

The previous paragraphs discussed the performance of different features characterising a scream. However, the automated scream detection considered a combination of these features, because no single feature defines a scream. This was demonstrated in Fig 6: which shows FPR declined faster than TPR when the number of votes required increased. Furthermore, screams did not need to conform to all feature thresholds, only to the number of required votes. However, the most salient features can be derived from Table 3. The event power and formant feature 5 defined screams most clearly, because these have the highest difference between votes for TPs and other sounds, of 69% and 76%, respectively. Furthermore these features account for at least 87% of the TPs. Other salient features include the event duration and formant feature 6 and 7 as they account for 91%, 93% and 93% of the TPs, respectively.

### Conclusion

This paper investigated which sound features define a pig scream in a pig barn. A classifier was constructed with a deliberate focus on explicit rules based and features with physical meaning. The resulting classifier had 71.83% sensitivity, 91.43% specificity and 83.61% precision. According to the classifier, a scream should have a high sound power, a formant structure and a certain duration. Two properties of lesser importance were the high frequency content and the variability of the signal. Furthermore, it was not necessary for a scream to have all these properties.
